# Effects of Cryopreservation on Cell Metabolic Activity and Function of Biofabricated Structures Laden with Osteoblasts

**DOI:** 10.3390/ma13081966

**Published:** 2020-04-22

**Authors:** Laura G. Hernández-Tapia, Zdenka Fohlerová, Jan Žídek, Marco A. Alvarez-Perez, Ladislav Čelko, Jozef Kaiser, Edgar B. Montufar

**Affiliations:** 1 CEITEC—Central European Institute of Technology, Brno University of Technology, Purkynova 123, 612 00 Brno, Czech Republic; 2 Tissue Bioengineering Laboratory, Faculty of Dentistry, Universidad Nacional Autónoma de México, Coyoacan, Mexico City 04510, Mexico

**Keywords:** biofabrication, bioprinting, cryopreservation, bone construct, osteoblast, metabolic activity, ALP activity

## Abstract

Biofabrication and maturation of bone constructs is a long-term task that requires a high degree of specialization. This specialization falls onto the hierarchy complexity of the bone tissue that limits the transfer of this technology to the clinic. This work studied the effects of the short-term cryopreservation on biofabricated osteoblast-containing structures, with the final aim to make them steadily available in biobanks. The biological responses studied include the osteoblast post-thawing metabolic activity and the recovery of the osteoblastic function of 3D-bioprinted osteoblastic structures and beta tricalcium phosphate (β-TCP) scaffolds infiltrated with osteoblasts encapsulated in a hydrogel. The obtained structures were cryopreserved at −80 °C for 7 days using dimethyl sulfoxide (DMSO) as cryoprotectant additive. After thawing the structures were cultured up to 14 days. The results revealed fundamental biological aspects for the successful cryopreservation of osteoblast constructs. In summary, immature osteoblasts take longer to recover than mature osteoblasts. The pre-cryopreservation culture period had an important effect on the metabolic activity and function maintain, faster recovering normal values when cryopreserved after longer-term culture (7 days). The use of β-TCP scaffolds further improved the osteoblast survival after cryopreservation, resulting in similar levels of alkaline phosphatase activity in comparison with the non-preserved structures. These results contribute to the understanding of the biology of cryopreserved osteoblast constructs, approaching biofabrication to the clinical practice.

## 1. Introduction

In 2016, Groll et al. described biofabrication in the context of tissue engineering and regenerative medicine as the application of automatic manufacturing processes to generate cell-biomaterial constructs that through the spatial arrangement of their components may mature into functional tissue equivalents [1]. This description is closely related to the aim of tissue engineering to develop biological substitutes that restore, maintain or improve the structure and function of damaged tissues or organs [2]. Biofabricated cell-material constructs aim to mimic tissue specific hierarchical features, consequently, their use is not limited to regenerative medicine applications, but they are also used as complex three-dimensional (3D) in vitro models for the study of developmental biology [3,4], cell biology [5,6], personalized pharmacokinetics [7] and disease pathogenesis [8,9]. In practice, biofabrication involves the structural aggregation of cells, materials, extracellular matrix components, bioactive molecules and possibly pre-biofabricated building blocks to control the fate of the stem cells [10,11]. Biofabrication should benefit from the computer-aided design, but it can use the original tissue engineering approach, where cells are seeded and cultured onto the scaffold, which in turn was produced either by additive manufacturing or by traditional casting or subtractive manufacturing approaches.

Despite bone tissue has a significant reparative capacity, more than two million grafts are performed annually to treat bone defects caused by degenerative diseases, accidents, tumours, infections and congenital diseases [12,13]. Therefore, bone regenerative medicine is one of the fields where biofabrication could provide an alternative that satisfies the increasing demand for bone grafts. However, the biofabrication of functional bone substitutes is a long task that requires specialization due to the hierarchical complexity of the bone tissue, restricting the transfer of this technology towards the clinic and hinders its routinely use to study fundamental aspects of bone biology. The cryopreservation of biofabricated bone substitutes is an interesting alternative to promote their transfer towards clinical and experimental practices. By cryopreservation, it will be possible to store ready-to-use bone substitutes in biobanks and their on-demand transportation, escaping in situ manufacturing and long maturation processes. The lasts could be performed in specialized laboratories following good manufacturing practices, therefore, fulfilling international standards.

Cryopreservation is the use of very low temperature to preserve living cells and tissues in a quiescent status for a long period, without losing their viability, activity, and function [14]. Several works have been devoted to the cryopreservation of engineered biological substitutes [15,16,17,18,19,20,21,22,23,24,25], but there is still the need for information on the cryopreservation of biofabricated osteoblast constructs, a paramount goal addressed in this work. Pioneer studies demonstrate that post-thawing osteoblast viability was better maintained (40–50%) when cryopreserved in dimethyl sulfoxide (DMSO) than in other cryoprotectant additives [26,27]. Moreover, it was reported that post-thawing cell viability was reduced for attached osteoblasts and increased with osteoblast density [27]. Unlike those early studies concentrated in the osteoblast post-thawing viability due to the use of different cryoprotectant additives under traditional two-dimensional (2D) cell culture, this work aims to evaluate the recovery of the metabolic activity and function of 3D-bioprinted osteoblast-containing structures and hydrogel-encapsulated osteoblasts infiltrated in beta-tricalcium phosphate (β-TCP) scaffolds; both freshly biofabricated structures were named osteoblast constructs for the sake of simplicity. Among the different alternatives, a hydrogel mixture composed of gelatine with alginate was selected for the encapsulation and 3D-bioprinting of osteoblasts. This hybrid hydrogel is easily processed into 3D structures, shows short-term stability after ionic crosslinking, supports cell proliferation in vitro, promotes better cell metabolic activity than pure alginate and supports bone healing in vivo [28,29,30,31,32]. Besides, the rationale of the use of β-TCP scaffolds was to mimic the chemical and mechanical microenvironment in bone tissue and, therefore, increase the structural properties of the constructs for better long-term in vitro manipulation. Variables such as the osteoblast maturity phenotype and the pre-cryopreservation culture period were studied for the first time and compared with the results of non-preserved counterparts. The short-term cryopreservation at −80 °C was studied as the proof of concept, discovering important aspects for the successful cryopreservation of osteoblast constructs.

## 2. Materials and Methods

### 2.1. Cell Culture

The study was performed using two different human osteoblastic cell lines, MG-63 (ATCC^®^ CRL-1427™, Manassas, VA, USA) and Saos-2 (ATCC^®^ HTB-85™, Manassas, VA, USA), maintained in Dulbecco’s Modified Eagle Medium (DMEM, Corning, NY, USA) supplemented with 10% Foetal Bovine Serum (FBS, Corning NY, USA) and antibiotics (streptomycin 100 μg/mL, penicillin 100 UI/mL and fungizone 0.3 μg/mL, Sigma-Aldrich, St. Louis, MS, USA). Despite the two cell lines having an osteosarcoma origin they are commonly used as an osteoblastic in vitro model in experiments that require large numbers of cells, generating reproducible results [33,34]. For all the experiments the cell lines were used at 3–6 passages after standard cryopreservation in liquid nitrogen, using DMSO (Sigma-Aldrich, St. Louis, MS, USA) as cryoprotectant, and 2 independent biological replicates with 3 samples per test were used for statistical analysis. The cultures were incubated at 37 °C in a 100% humidified atmosphere with 5% CO_2_ and medium renewal every 3 days.

### 2.2. Bioprinting and Culture of Osteoblast Constructs

The ink for cell printing was optimized following the methods described previously [28,29,30,35] and consisted of 10 wt.% sterile gelatine (Bovine skin type-B, Bloom number of 225 g, Sigma-Aldrich, St. Louis, MS, USA) and 2 wt.% sterile sodium alginate (120,000–190,000 g/mol and M/G ratio of 1.56, Sigma-Aldrich, St. Louis, MS, USA) dissolved in FBS. The ink was mixed under sterile conditions in a dual asymmetric centrifugal mixer (DAC 150, Speedmixer, Hamm, Germany) at 3500 rpm for 5 min at 60 °C. Despite the short mixing time, the temperature used may have led to the partial heat-inactivation of FBS. However, it was not considered a disadvantage because several cell culture studies are performed with heat-inactivated FBS and the fact that the cell culture medium contains fresh FBS. Afterwards, MG-63 or Saos-2 cells at 95% of confluence were detached from the cell culture flask using Trypsin-EDTA solution (Sigma-Aldrich, St. Louis, MS, USA) and the cells were centrifuged and suspended in the ink at 37 °C at a density of 1 × 10^6^ cells/mL. Then, 3 mL of the bioink (ink containing osteoblast) was transferred into the cartridge of the robotic dispensing device (Pastecaster, Fundació CIM, Barcelona, Spain) and the osteoblast constructs were fabricated by extrusion under sterile conditions, i.e., placing the device inside a laminar flow cabinet and sterilising all the materials in direct contact with the cells by autoclave prior to bioprinting. The Pastecaster robot is not equipped with a heating system, therefore, the fabrication was started when the flow of the bioink at the outlet of the dispensing nozzle (250 µm SmoothFlow tapered tip, Nordson EFD, East providence, RI, USA) was visually suitable for printing, i.e., the bioink filament was continuous and does not flow when deposited on the printing platform. Presumably, this condition was achieved when the temperature of the bioink in the cartridge was 25 °C [35] and was maintained long enough to extrude all the bioink. The room temperature in the cell culture lab was 22 °C.

A G-code was digitally designed in MATLAB (Version 9.4.0.813654, The MathWorks, Inc., Natick, MA, USA) to deposit automatically one construct of 12 mm in diameter by 1 mm in height in each well of a standard 24-well cell culture plate. The parameters for 3D-bioprinting were set as follows: cartesian grid pattern with a distance between filaments of 500 µm to allow mass transport, four layers of 250 µm of height, printing speed of 10 mm/s and pressure of 110 kPa. After fabrication, each construct was immersed in 50 µL of CaCl_2_ solution (100 mM, Lach-Ner, Neratovice, Czech Republic) at 10 °C for the ionic cross-linking of alginate, increasing the structural properties of the constructs [30]. Immediately, 500 µL of cell culture medium preheated at 37 °C was added and the constructs were maintained at 37 °C for up to 7 days.

Cell viability after bioprinting was determined by a live/dead viability kit (Invitrogen, Waltham, MA, USA). Briefly, the samples were rinsed in PBS and incubated for 15 min with 2 µM Calcein-AM and 1.5 µM ethidium homodimer-1 solution in PBS and finally rinsed twice in PBS before imaging with a fluorescent microscope coupled to a CCD camera (Zeiss, Oberkochen, Germany), using appropriate filter sets. Cell viability was measured via counting live (green) and dead (red) cells using ImageJ software (National Institutes of Health, Bethesda, MD, USA). The cell metabolic activity was evaluated by XTT assay (Section 2.6) and the osteoblastic phenotype was evaluated by the alkaline phosphatase (ALP) activity (Section 2.7) and Alizarin red S staining (Section 2.8).

### 2.3. Additive Manufacturing of Beta Tricalcium Phosphate Scaffolds

Commercial β-TCP powder (GPR Rectapur VWR, BDH Prolabo, Leuven, Belgium) with a median particle size of 2.3 µm and Poloxamer 407 (Sigma-Aldrich, St. Louis, MS, USA) were used to prepare the ceramic ink for robocasting as described by Casas-Luna et al., [36]. Briefly, 3 g of β-TCP powder was mixed with 3 g of Poloxamer 407 aqueous solution (40 wt.%) to obtain the ink that was transferred into the cartridge (3cc Optimum^®^ Syringe Barrels, Nordson EFD, East providence, RI, USA) of the robotic deposition device (Pastecaster, Fundació CIM, Barcelona, Spain). Immediately, 5-layer scaffolds with 11 mm in diameter and 2 mm in height were deposited at 22 °C at a printing rate of 8 mm/s, using cartesian grid pattern and a tapered dispensing nozzle of 410 µm (SmoothFlow Tapered Tips, Nordson EFD, East providence, RI, USA). The nominal pore size in the printing plane was set to 400 µm. The extrusion force was adjusted to maintain a steady extrusion rate matching the printing rate. After drying at room temperature the scaffolds were sintered at 1200 °C for 5 h. Representative images of the microstructure of the scaffolds were obtained with a scanning electron microscope (SEM; TESCAN Lyra3, Brno, Czech Republic), using an electron beam voltage of 5 kV in carbon-coated samples. Finally, the scaffolds were sterilized by gamma irradiation at 25 KGy prior to perform cell cultures.

### 2.4. Cell Culture in Beta Tricalcium Phosphate Scaffolds

Only mature osteoblastic phenotype cells (Saos-2) were used for the evaluation of the 3D-culture in β-TCP scaffolds. Briefly, Saos-2 cells were suspended at 37 °C at a density of 1.5 × 10^6^ cells/mL in a hydrogel solution composed of 3 wt.% gelatine and 2 wt.% sodium alginate in FBS. In this case, a high stiffness hydrogel is not required, as the structural stability of the construct will be provided by the β-TCP scaffold. Consequently, the gelatine concentration was reduced in comparison with the bioink for cell printing (Section 2.2). One drop of the cell suspension (100 µL) was added to each scaffold for osteoblasts infiltration and immediately was crosslinked with 50 µL of CaCl_2_ solution (100 mM) at 10 °C. Afterwards, 500 µL of cell culture medium preheated at 37 °C was added and the constructs were maintained at 37 °C for up to 21 days. Note that the cell density of the suspension was increased in order to have similar initial cell density in the construct than in 3D-bioprinting. The cell metabolic activity was evaluated by XTT assay (Section 2.6) and the osteoblastic phenotype was evaluated by the ALP activity (Section 2.7).

### 2.5. Cryopreservation of the Osteoblast Constructs

3D-bioprinted osteoblast constructs and β-TCP scaffolds infiltrated with encapsulated osteoblasts were subject to the cryopreservation protocol after 3 and 7 days of culture. Such points were selected to allow the recovery of the cell viability after biofabrication and promote cell–cell interactions within the constructs. Briefly, after being washed two times with PBS at 37 °C, the samples were transferred to a new 24-well cell culture plate and covered with 500 µL of 20% DMSO (Sigma-Aldrich, St. Louis, MS, USA) at 4 °C diluted in cell culture medium. Immediately the plate containing the samples was frozen at a cooling rate of 1 °C/min up to −50 °C (Epsilon 2-10D LSCplus, Christ, Osterode am Harz, Germany) and finally stored in a freezer at −80 °C (ProfiMaster PMU0485, National Lab GmbH, Moelln, Germany). For the evaluation of the cell metabolic activity (Section 2.6) and the ALP activity (Section 2.7) after cryopreservation the samples were thawed by adding 500 µL of cell culture medium at 37 °C. Immediately after samples defrosted the medium was removed; the samples were washed two times with cell culture medium and continued their culture up to 14 days at 37 °C in 500 µL of medium. The biological assays referred were performed immediately after thawing and after 7 and 14 days of effective culture, i.e., without considering the period of cryopreservation. Results were compared with the outcomes of the counterparts cultured for the same periods but non-cryopreserved.

### 2.6. Cell Metabolic Activity

Cell metabolic activity was evaluated with and without the cryopreservation of the constructs by XTT assay (Thermo Fisher Scientific Inc., Waltham, MA, USA). The assay is based on the cleavage of the tetrazolium salt XTT (2,3-Bis-(2-Methoxy-4-Nitro-5-Sulfophenyl)-2H-Tetrazolium-5-Carboxanilide) in the presence of an electron-coupling reagent, producing a soluble formazan salt. The concentration of the formazan product is directly proportional to the number of metabolically active cells. Briefly, constructs were washed three times with PBS and then transferred to a new cell culture plate to discard the cells that could be attached to the bottom of the wells. Afterwards, the constructs were incubated at 37 °C for 2 h with 500 µL of the XTT labelling mixture (0.4 mg/mL) in complete cell culture medium. Then, 150 µL of the supernatant per sample was transferred to a 96-well cell culture plate and the optical absorbance (OA) was quantified by spectrophotometry at 450 nm with a plate reader. Results are reported as relative fold change in comparison with OA measured after 3 days of cell culture without cryopreservation.

### 2.7. Alkaline Phosphatase Activity

The ALP activity was evaluated with and without cryopreservation of the constructs by an ALP colorimetric assay kit (Abcam plc, Cambridge, UK). Before the assay, cell culture medium was removed, the constructs were washed three times with PBS and lysed using 400 µL of 0.1% Triton X-100 in PBS for 10 min at 37 °C. All the lysates were centrifuged at 4 °C for 15 min at 15000 rpm to remove solid particles. To perform the ALP assay, 80 µL of lysate was incubated for 1 h at room temperature in a 96-well plate with 50 µL of 5 mM p-nitrophenyl phosphate (pNPP) solution. Afterward, the reaction was stopped with 20 µL of stop solution and the OA was quantified by spectrophotometry at 405 nm with a plate reader. Three replicates per sample were tested. The ALP activity was calculated as the p-nitrophenol (pNP) generated by samples during the incubation per unit of sample volume per minute of reaction. The assay is based on the dephosphorylation of pNPP by the ALP enzyme generating a yellow colour. A standard curve was produced with the ALP enzyme provided in the assay kit, which reacted with serial dilutions of pNPP.

### 2.8. Alizarin red S Staining

The recovery of the mature osteoblast function after cryopreservation was determined by visual detection of cell-mediated deposition of calcium salts using Alizarin red staining. Briefly, 3D-bioprinted cryopreserved constructs were washed two times with PBS after 7 days of culture, then incubated with 40 mM Alizarin red S solution (Sigma-Aldrich, St. Louis, MS, USA) for 30 min at room temperature, thoroughly washed with distilled water to remove the excess of Alizarin dye and photographed using a stereomicroscope (Olympus DSX-HRSU, Tokyo, Japan). 3D-cultures in β-TCP scaffolds were not stained due to the chemical nature of calcium phosphates that intensely interact with Alizarin red.

### 2.9. Cell Morphology in Tricalcium Phosphate Scaffolds

The morphology of the cells encapsulated in the hydrogel infiltrated in the β-TCP scaffolds was examined by confocal microscopy (Axio Observer.Z1 with confocal unit LSM 800, Zeiss, Oberkochen, Germany). Briefly, the samples were washed three times with PBS, fixed with 4% paraformaldehyde solution for 15 min at 4 °C and then washed with PBS. The nuclei of cells were stained with DAPI (300 nM; Invitrogen, Waltham, MA, USA) at 25 °C for 5 min and fluorescent images (358/461 nm) were acquired in parallel with widefield (WF) images.

### 2.10. Statistical Analysis

All statistical analyses were performed using Sigma Plot software (Version 12, Systat Software Inc., San Jose, CA, USA). The data presented correspond to the mean ± standard deviation (n = 3). Data were evaluated by two-way analysis of variance followed by Turkey’s multiple comparison tests. Statistical significance was observed when compared cryopreserved and non-preserved samples at the same time point and same samples at different experimental points at a level of *p < 0.05, **p < 0.01, ***p < 0.001.

## 3. Results

### 3.1. Bioprinted Osteoblast Constructs

**Figure 1:**
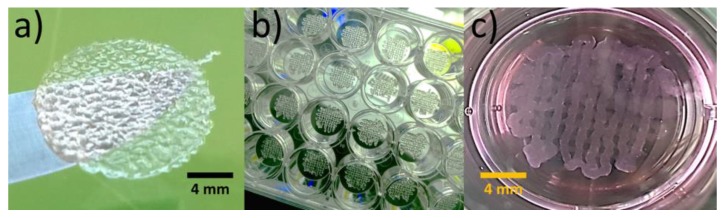
Representative images of 3D-bioprinted osteoblast constructs: (**a**) construct fully swelled, (**b**) 24 constructs automatically bioprinted in the cell culture plate and (**c**) construct immersed in cell culture medium at 37 °C after crosslinking with CaCl_2_.

Figure 1 shows that 3D-bioprinted osteoblast constructs were structurally stable after crosslinking with CaCl_2_ solution. Both, MG-63 and Saos-2 constructs showed initial cell viability of 80%. The automatic deposition allowed the fabrication of osteoblast constructs with identical shape directly in the 24-well cell culture plate (Figure 1b), reducing the risk of samples contamination. Moreover, the constructs reproduced with fidelity the cartesian pattern after 3D-bioprinting (Figure 1b) and retained the architecture after immersion in cell culture medium at 37 °C (Figure 1c).

### 3.2. Culture and Cryopreservation of Bioprinted Osteoblast Constructs

**Figure 2:**
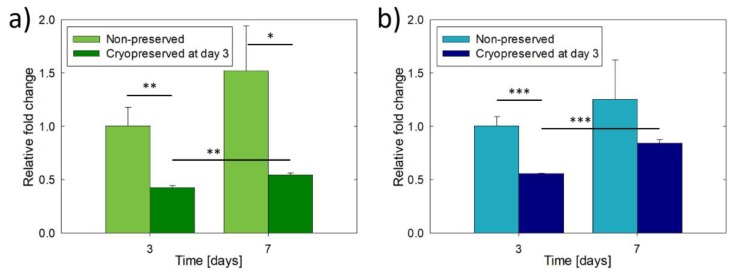
Cell metabolic activity of 3D-bioprinted osteoblast constructs with or without cryopreservation after 3 days of culture: (**a**) MG-63 constructs and (**b**) Saos-2 constructs. The results are expressed as relative fold change compared to the cell metabolic activity obtained on day 3 without cryopreservation. Asterisks indicate statistically significant differences at *p < 0.05, **p < 0.01, ***p < 0.001, n = 3.

Figure 2 shows the cell metabolic activity of the MG-63 and Saos-2 constructs with and without cryopreservation. In particular, the cryopreserved constructs were frozen at day 3 of culture and after thawing were cultivated for a total of 7 days. The cell metabolic activity of the non-preserved constructs increased more than 1.3 times between 3 and 7 days of culture, however, without showing statistically significant differences due to the dispersion in the results. The cultures were not maintained longer than 7 days because, similar to previous works, the constructs loosed their structural integrity due to the progressive dissolution of uncrosslinked gelatine [29,30]. The two types of osteoblast constructs were metabolically active after cryopreservation, but the metabolic activity was reduced to the half of the non-preserved constructs (day 3). MG-63 constructs showed a quiescent phase after cryopreservation, showing 1.3 times the cell metabolic activity of day 3 and only reaching 36% of activity of the non-preserved constructs at day 7. In contrast, Saos-2 cryopreserved constructs recovered faster their metabolic activity, showing no statistically significant differences in comparison to non-preserved constructs at day 7 and 1.5 times higher activity than at day 3.

**Figure 3:**
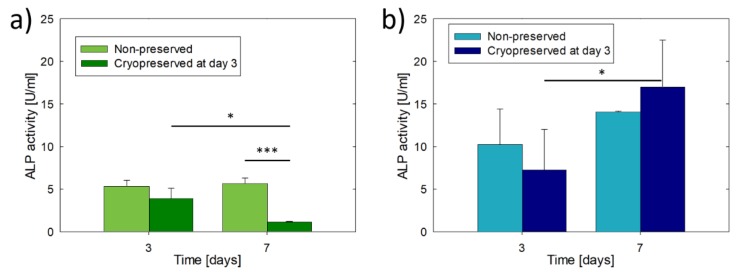
Alkaline phosphatase (ALP) activity of 3D-bioprinted osteoblast constructs with or without cryopreservation after 3 days of culture: (**a**) MG-63 constructs and (**b**) Saos-2 constructs. Asterisks indicate statistically significant differences at *p < 0.05, ***p < 0.001, n = 3.

The differences between MG-63 and Saos-2 constructs were also observed in terms of ALP activity (Figure 3). Without cryopreservation, the ALP activity of MG-63 constructs did not change over the culture time, while it increased 1.4 times in Saos-2 constructs. However, the increment was not statistically significant. In general, the ALP activity of Saos-2 constructs was two times higher than that of MG-63 constructs due to the more mature osteoblastic phenotype. No statistically significant differences in ALP activity were observed at day 3 within the same type of construct due to cryopreservation (*p* = 0.177 for MG-63 and *p* = 0.577 for Saos-2). The cryopreserved MG-63 constructs showed a five-fold reduction in ALP activity after 7 days of culture, while the cryopreserved Saos-2 constructs showed higher ALP activity, but not statistically significant (*p* = 0.315), than their non-preserved counterparts, indicating in this case the recovery of the osteoblast function.

**Figure 4:**
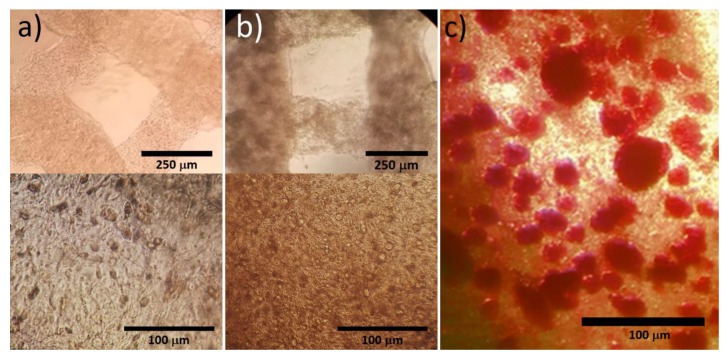
Optical microscope images of 3D-bioprinted osteoblast constructs after cryopreservation: (**a**) representative MG-63 construct, (**b**) representative Saos-2 construct and (**c**) positive Alizarin red S staining of a Saos-2 construct, showing in red colour the salts of calcium minerals. The samples were cryopreserved at day 3 of culture and the images were obtained after 7 days of culture.

The recovery of the osteoblastic function of the cryopreserved Saos-2 constructs is also observed in the optical microscope images in Figure 4. MG-63 constructs appeared visually clearer (Figure 4a) than Saos-2 constructs (Figure 4b) after 7 days of culture. The darker aspect of the cryopreserved Saos-2 constructs was due to the deposition of calcium mineral salts, as confirmed by the positive Alizarin red S staining (Figure 4c). The two types of constructs contain rounded cells distributed over the whole structure (Figure 4a,b). The regions rich in calcium minerals stained with Alizarin red S have globular morphology and are bigger than individual cells.

### 3.3. Culture of Hydrogel Encapsulated Saos-2 Cells in β-TCP Scaffolds

**Figure 5:**
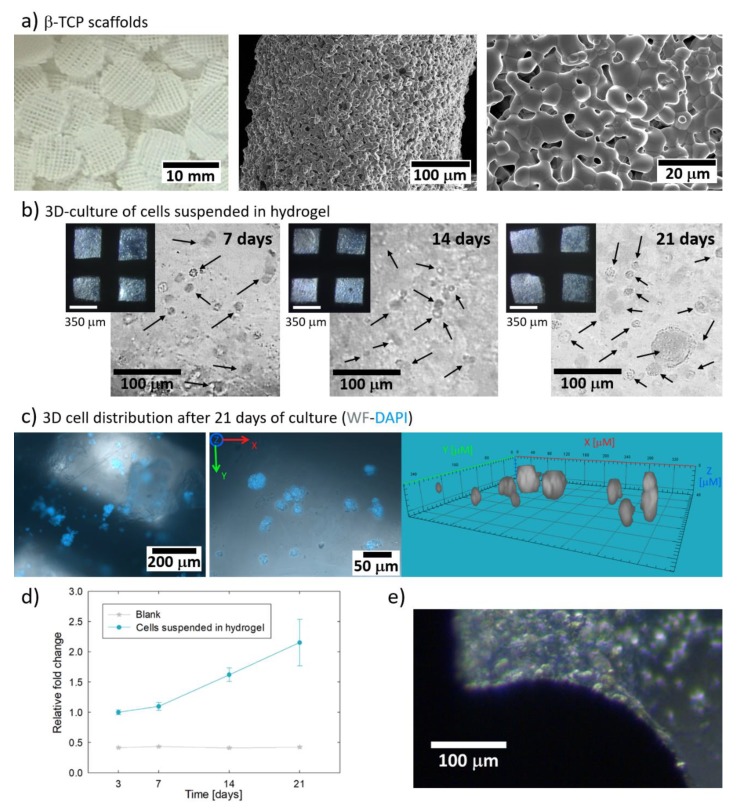
Culture of cells encapsulated in hydrogel inside beta tricalcium phosphate (β-TCP) scaffolds: (**a**) shape and microstructure of the β-TCP scaffolds without cells, (**b**) images of the encapsulated Saos-2 cells growing in globular aggregates (pointed by arrows) inside β-TCP scaffolds, (**c**) confocal microscope image stack (widefield (WF) (grey) and DAPI (blue)) and 3D virtual reconstruction of the cell aggregates after 21 days of culture, (**d**) cell metabolic activity expressed as relative fold change compared to the cell metabolic activity obtained on day 3 (n = 3), and (**e**) example of cells lining the surface of the scaffold after 14 days of culture.

The β-TCP scaffolds with cartesian grid pattern were successfully fabricated by robocasting (Figure 5a). The macropore size in the longitudinal direction (printing plane) was close to ~350 µm instead than nominal 400 µm due to the shrinkage of the scaffolds during sintering. Furthermore, Figure 5a shows that the strands of the scaffolds have a microstructure of smooth polyhedral grains and 15% of micropores, around 1 µm in size. The porous network allowed the easy infiltration of the cell-loaded hydrogel inside the scaffolds. Moreover, the use of β-TCP scaffolds removed the problem of structural integrity loosening, allowing the culture and manipulation of the osteoblast constructs for at least 21 days (Figure 5b). Despite the opacity of β-TCP scaffolds, the translucence of the hydrogel allowed the imaging of the cells through the open macropores of the scaffold. The cells grew in globular aggregates with size between 50 and 80 µm, homogeneously distributed inside the macropores (Figure 5b), as corroborated by the fluorescent staining of the cell nuclei (Figure 5c). Furthermore, the cell metabolic activity increased over time (Figure 5d), corroborating the cytocompatibility of the hydrogel infiltrated in the scaffolds. It was not evident the cell migration towards the surface of the scaffolds, but occasionally some cells were observed lining some areas of the surface of the strands (Figure 5e).

### 3.4. Cryopreservation of Hydrogel Encapsulated Saos-2 Cells Cultured in β-TCP Scaffolds

**Figure 6:**
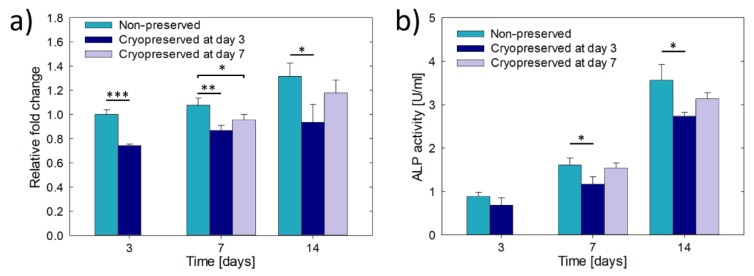
Results of cryopreservation of Saos-2 cells encapsulated in hydrogel and infiltrated in β-TCP scaffolds: (**a**) cell metabolic activity expressed as relative fold change compared to the cell metabolic activity obtained at day 3 without cryopreservation, and (**b**) ALP activity. Asterisks indicate statistically significant differences at *p < 0.05, ** *p* < 0.01, *** *p* < 0.001, n = 3.

Figure 6 shows the cell metabolic activity and the ALP activity of osteoblast constructs consisting of hydrogel encapsulated Saos-2 cells infiltrated in β-TCP scaffolds. Both, the cell metabolic activity and the ALP activity, with and without cryopreservation, increased through the culture time, showing that though the activities were reduced, the constructs were metabolically active and retained the osteoblastic phenotype after cryopreservation. The lowest activities were observed for the constructs frozen at day 3 of pre-culture, always showing values statistically below the non-preserved constructs. In other words, they did not have enough time to fully recover. In contrast, when the constructs were cryopreserved after 7 days of pre-culture, both, the cell metabolic activity and the ALP activity were between the values obtained for non-preserved constructs and constructs cryopreserved at day 3. Moreover, at day 14 the two activities did not show statistically significant differences with respect to the non-preserved constructs. Therefore, the osteoblast constructs entirely recovered the activity and function. Lastly, no construct presented signals of structural damage due to thermal shock during cryopreservation.

## 4. Discussion

The effects of the cryopreservation on the biological behaviour of osteoblasts (MG-63 and Saos-2) were studied either in 3D-bioprinted cell containing structures or in cells encapsulated in a hydrogel inside of β-TCP scaffolds. In general, the results prove the feasibility of cryopreserving the osteoblast constructs following a simple and low-cost methodology equivalent to that used for the cryopreservation of cells [37] and human embryos [38]. The primary challenge for the sucesfull cryopreservation of osteoblast constructs is to avoid the death of the cells due to cryoinjury produced by intracellular ice formation and cell dehydration [39,40]. The growth of ice crystals is suppressed by reducing the cooling rate during freezing. It is well accepted that cooling rates around 1 °C/min are effective for cryopreservation of cells [37]. However, such a slow cooling rate allows the dehydration of the cells leading to their death due to osmotic shock. Thus, permeating or non-permeating cryoprotectant additives are required to avoid cell dehydration. DMSO was used in this work as a cryoprotectant additive because it is traditionally the common cryoprotectant used in cell biology laboratories [37,41]. DMSO also offers the advantage of permeating deep into tissues rapidly [37], thus, (as uncovered in this work) allowing the protection of the cells in 3D, deep inside the hydrogels used for biofabrication.

The results of this work reveal important aspects that must be considered for the successful cryopreservation of osteoblast constructs. For example, mature phenotype osteoblasts, such as mineral forming Saos-2 cells, recovered their cell metabolic activity, ALP activity and the ability to deposit nodules of mineralization after the cryopreservation faster than pre-osteoblasts or immature osteoblasts, such as MG-63 cells. The reasons for these observations are still unknown and further studies are required to understand the reasons for the loss of function of immature osteoblasts. Furthermore, and in agreement with previous studies on the cryopreservation of hepatocytes and human embryonic stem cells [22,42], the pre-cryopreservation culture period has important effects on the metabolic activity and function maintain by osteoblasts, which recover normal values faster when cryopreserved after longer-term of culture (7 days) than at shorter-term (3 days). The production of the bone extracellular matrix probably protects the osteoblasts from cryoinjury. In fact, macromolecules such as proteins in FBS or the extracellular matrix, act as cryoprotectant additive by reducing the freezing point of DMSO, therefore minimizing the risk of intracellular ice formation, and also protect the cells from dehydration [43,44]. Another benefit of longer culture before cryopreservation is the formation of more cell–cell and cell–extracellular matrix adhesions that have been shown to reduce cryoinjury [20,42,45]. The use of β-TCP scaffolds further improves the osteoblast survival after cryopreservation, which in turn results in similar levels of ALP activity in comparison with the non-preserved constructs. Although the real factors responsible for this are unknown, it is hypothesized that the microstructure of the scaffold protect the cells better from dehydration through a capillary force related mechanism. Finally yet importantly, the stiff β-TCP scaffolds provide the load-bearing capacity to the osteoblast construct, without compromising the viability of the cells. Despite not being measured in this study, the compressive strength of β-TCP scaffolds range from 1 to 20 MPa [36,46], three orders of magnitude above the stiffness of hydrogels; this is an important advantage for the structural integrity and improved manipulation of the osteoblast constructs. Furthermore, the β-TCP scaffolds may provide a microenvironment similar to that of the natural bone. Previous studies showed that the ionic interactions of calcium phosphate ceramics with the physiological environment could promote the differentiation of osteoblasts or mesenchymal stromal cells into the osteoblastic phenotype [47,48,49].

Although the cryopreservation of ready-to-use bone constructs is desirable, for the application in practice there is still the need to study long-term cryopreservation at a lower temperature than −80 °C and how the gene expression is up or down regulated due to cryopreservation. Another issue to be studied is the difference in phenotype between osteosarcoma cell lines and primary osteoblasts [50]. In general, the lower proliferation rate and contact inhibition growth of primary osteoblasts could make more challenging their successful cryopreservation. Preliminary results show a 50% survival rate of rabbit-derived primary osteoblasts in comparison with 80% of Saos-2 cells [26]. 

While for a 3D in vitro model it is enough to have a reproducible cryopreservation method that does not damage the cells, neither compromise their activity and function after thawing, more demanding requirements arise for the clinical applications [16,40]. One concern is the use of FBS either as a bioink or as cryoprotectant supplement since it introduces the risk of immunological rejection and the transmission of diseases. The alternative is the use of xeno-free cryoprotectant supplements [51,52]. Moreover, for the implementation of autologous cell therapies, there is still the need for primary cell extraction and expansion, making more challenging the treatment of bone injuries that require immediate attention.

When tackling all these issues will be possible to offer the bone constructs off-the-shelf all over the world. The distribution of on-demand manufactured constructs ought to be less expensive and less time consuming than their in situ biofabrication, providing this technology to a greater number of researchers and clinicians not engaged in biofabrication but with other relevant scientific interests. It is predicted that this will spread the safe and reliable use of biofabricated bone constructs, either as in vitro bone tissue models or as bone grafts in regenerative medicine.

An alternative route to the described methodology is the cryopreservation of the osteoblast constructs by vitrification, a non-equilibrium freezing technique that results in a glass formation instead of growth of ice crystals through the combination of high concentration of cryoprotectant additive and ultrarapid freezing [17,18].

## 5. Conclusions

This work shows that the short-term cryogenic preservation of osteoblast constructs is feasible in DMSO at −80 °C. The results uncover that the cell metabolic activity and function of the osteoblast constructs after thawing strongly depend on the maturity of the osteoblasts, the pre-cryopreservation culture period and the use of β-TCP scaffolds. In general, the faster recovery was achieved when mature osteoblast constructs were cryopreserved after long periods of pre-culture, preferably with the use of β-TCP scaffolds. The β-TCP scaffolds also provided structural integrity to the constructs, resulting in better handling and shape retention for a longer time. Since cell recovery depends on the osteoblast maturity, there is still the need to assess the effects of cryopreservation on primary osteoblast constructs, promoting the use of xeno-free cryoprotectant additives.

## References

[1] Groll J., Boland T., Blunk T., Burdick J.A., Cho D.W., Dalton P.D., Derby B., Forgacs G., Li Q., Mironov V.A. (2016). Biofabrication: Reappraising the definition of an evolving field. Biofabrication.

[2] Langer R., Vacanti J.P. (1993). Tissue engineering. Science.

[3] Marga F., Neagu A., Kosztin I., Forgacs G. (2007). Developmental biology and tissue engineering. Birth Defects Res. Part C Embryo Today Rev..

[4] Gu Q., Tomaskovic-Crook E., Lozano R., Chen Y., Kapsa R.M., Zhou Q., Wallace G.G., Crook J.M. (2016). Functional 3D Neural mini-tissues from printed gel-based bioink and human neural stem cells. Adv. Healthc. Mater..

[5] Murphy S.V., Atala A. (2014). 3D bioprinting of tissues and organs. Nat. Biotechnol..

[6] Tasoglu S., Demirci U. (2013). Bioprinting for stem cell research. Trends Biotechnol..

[7] Xu F., Wu J., Wang S., Durmus N.G., Gurkan U.A., Demirci U. (2011). Microengineering methods for cell-based microarrays and high-throughput drug-screening applications. Biofabrication.

[8] Samavedi S., Joy N. (2017). 3D printing for the development of in vitro cancer models. Curr. Opin. Biomed. Eng..

[9] Taubenberger A.V. (2014). In vitro microenvironments to study breast cancer bone colonisation. Adv. Drug. Deliv. Rev..

[10] Mir T.A., Nakamura M. (2017). Three-Dimensional Bioprinting: Toward the era of manufacturing human organs as spare parts for healthcare and medicine. Tissue Eng. Part B Rev..

[11] Raman R., Bashir R. (2017). Biomimicry, biofabrication, and biohybrid systems: The emergence and evolution of biological desigin. Adv. Healthc. Mater..

[12] Giannoudis P.V., Dinopoulos H., Tsiridis E. (2005). Bone substitutes: An update. Injury.

[13] Shegarfi H., Reikeras O. (2016). Review Article: Bone transplantation and immune response. J. Orthop. Surg..

[14] Hunt C.J. (2011). Cryopreservation of human stem cells for clinical application: A review. Transfus. Med. Hemotherapy.

[15] Pogozhykh O., Prokopyuk V., Prokopyuk O., Kuleshova L., Goltsev A., Figueiredo C., Pogozhykh D. (2018). Towards biobanking technologies for natural and bioengineered multicellular placental constructs. Biomaterials.

[16] Kuleshova L.L., Gouk S.S., Hutmacher D.W. (2007). Vitrification as a prospect for cryopreservation of tissue-engineered constructs. Biomaterials.

[17] Dahl S.L., Chen Z., Solan A.K., Brockbank K.G., Niklason L.E., Song Y.C. (2006). Feasibility of vitrification as a storage method for tissue-engineered blood vessels. Tissue Eng..

[18] Agudelo C.A., Iwata H. (2008). The development of alternative vitrification solutions for microencapsulated islets. Biomaterials.

[19] Malpique R., Osório L.M., Ferreira D.S., Ehrhart F., Brito C., Zimmermann H., Alves P.M. (2010). Alginate encapsulation as a novel strategy for the cryopreservation of neurospheres. Tissue Eng. Part C Methods.

[20] Ahmad H.F., Sambanis A. (2013). Cryopreservation effects on recombinant myoblasts encapsulated in adhesive alginate hydrogels. Acta Biomater..

[21] Zhao G., Liu X., Zhu K., He X. (2017). Hydrogel encapsulation facilitates rapid-cooling cryopreservation of stem cell-laden core-shell microcapsules as cell-biomaterial constructs. Adv. Healthc. Mater..

[22] Koebe H.G., Dunn J.C., Toner M., Sterling L.M., Hubel A., Cravalho E.G., Yarmush M.L., Tompkins R.G. (1990). A new approach to the cryopreservation of hepatocytes in a sandwich culture configuration. Cryobiology.

[23] Umemura E., Yamada Y., Nakamura S., Ito K., Hara K., Ueda M. (2011). Viable cryopreserving tissue-engineered cell-biomaterial for cell banking therapy in an effective cryoprotectant. Tissue Eng. Part C Methods.

[24] Teo K.Y., De Hoyos T.O., Dutton J.C., Grinnell F., Han B. (2011). Effects of freezing-induced cell-fluid-matrix interactions on the cells and extracellular matrix of engineered tissues. Biomaterials.

[25] Chen F., Zhang W., Wu W., Jin Y., Cen L., Kretlow J.D., Gao W., Dai Z., Wang J., Zhou G. (2011). Cryopreservation of tissue-engineered epithelial sheets in trehalose. Biomaterials.

[26] Kofron M.D., Opsitnick N.C., Attawia M.A., Laurencin C.T. (2003). Cryopreservation of tissue engineered constructs for bone. J. Orthop. Res..

[27] Liu B.L., McGrath J. (2004). Vitrification solutions for the cryopreservation of tissue-engineered bone. Cell Preserv. Technol..

[28] Luo Y., Lode A., Akkineni A.R., Gelinsky M. (2015). Concentrated gelatin/alginate composites for fabrication of predesigned scaffolds with a favorable cell response by 3D plotting. RSC Adv..

[29] Zehnder T., Sarker B., Boccaccini A.R., Detsch R. (2015). Evaluation of an alginate–gelatine crosslinked hydrogel for bioplotting. Biofabrication.

[30] Duan B., Hockaday L.A., Kang K.H., Butcher J.T. (2013). 3D Bioprinting of heterogeneous aortic valve conduits with alginate/gelatin hydrogels. J. Biomed. Mater. Res. Part A.

[31] Xia Y., Mei F., Duan Y., Gao Y., Xiong Z., Zhang T. (2012). Bone tissue engineering using bone marrow stromal cells and an injectable sodium alginate/gelatin scaffold. J. Biomed. Mater. Res. Part A.

[32] Stancu I.C., Dragusin D.M., Vasile E., Trusca R., Antoniac I., Vasilescu D.S. (2011). Porous calcium alginate-gelatin interpenetrated matrix and its biomineralization potential. J. Mater. Sci.: Mater. Med..

[33] Richards R., Czekanska E., Hayes J., Stoddart M. (2016). In search of an osteoblast cell model for in vitro research. Eur. Cells Mater..

[34] Czekanska E.M., Stoddart M.J., Ralphs J.R., Richards R.G., Hayes J.S. (2014). A phenotypic comparison of osteoblast cell lines versus human primary osteoblasts for biomaterials testing. J. Biomed. Mater. Res. Part A.

[35] Billiet T., Gevaert E., De Schryver T., Cornelissen M., Dubruel P. (2014). 3D printing of gelatin methacrylamide cell-laden tissue-engineered constructs with high cell viability. Biomaterials.

[36] Casas-Luna M., Tan H., Tkachenko S., Salamon D., Montufar E.B. (2019). Enhancement of mechanical properties of 3D-plotted tricalcium phosphate scaffolds by rapid sintering. J. Eur. Cer. Soc..

[37] Freshney R.I. (2010). Cryopreservation. Culture of Animal Cells: A Manual of Basic Technique and Specialized Applications.

[38] Takeo T., Kondo T., Haruguchi Y., Fukumoto K., Nakagawa Y., Takeshita Y., Nakamuta Y., Tsuchiyama S., Shimizu N., Hasegawa T. (2010). Short-term storage and transport at cold temperatures of 2-cell mouse embryos produced by cryopreserved sperm. J Am. Assoc. Lab. Anim. Sci..

[39] Mazur P., Leibo S.P., Chu E.H. (1972). A two-factor hypothesis of freezing injury. Evidence from Chinese hamster tissue-culture cells. Exp. Cell. Res..

[40] Karlsson J.O., Toner M. (1996). Long-term storage of tissues by cryopreservation: Critical issues. Biomaterials.

[41] Whittingham D.G., Leibo S.P., Mazur P. (1972). Survival of Mouse Embryos Frozen to −196° and −269 °C. Science.

[42] Ji L., De Pablo L.L., Palecek S.P. (2004). Cryopreservation of adherent human embryonic stem cells. Biotechnol. Bioeng..

[43] Goh B.C., Thirumala S., Kilroy G., Devireddy R.V., Gimble J.M. (2012). Cryopreservation characteristics of adipose-derived stem cells: Maintenance of differentiation potential and viability. J. Tissue Eng. Regen. Med..

[44] Carroll J., Wood M.J., Whittingham D.G. (1993). Normal fertilization and development of frozen-thawed Mouse Oocytes: Protective action of certain macromolecules. Biol. Reprod..

[45] Sambu S., Xu X., Schiffter H.A., Cui Z.F., Ye H. (2011). RGDS-Fuctionalized alginates improve the survival rate of encapsulated embryonic stem cells during cryopreservation. Cryo-Letters.

[46] Miranda P., Saiz E., Gryn K., Tomsia A.P. (2006). Sintering and robocasting of beta-tricalcium phosphate scaffolds for orthopaedic applications. Acta Biomater..

[47] Varghese S., Chien S., Gianneschi N.C., Kang H., Vecchio K.S., Caro E.J., Phadke A., Theodorakis E.A., Siu M., Hwang Y. (2014). Calcium phosphate-bearing matrices induce osteogenic differentiation of stem cells through adenosine signaling. Proc. Natl. Acad. Sci. USA.

[48] Lee M.N., Hwang H.S., Oh S.O., Roshanzadeh A., Kim J.W., Song J.H., Kim E.S., Koh J.T. (2018). Elevated extracellular calcium ions promote proliferation and migration of mesenchymal stem cells via increasing osteopontin expression. Exp. Mol. Med..

[49] Wang P., Zhao L., Liu J., Zhou X., Weir M.D. (2014). Bone tissue engineering via nanostructured calcium phosphate biomaterials and stem cells. Bone Res..

[50] Pautke C., Schieker M., Tischer T., Kolk A., Neth P., Mutscher W., Milz S. (2004). Characterization of osteosarcoma cell lines MG-63, Saos-2 and U-2OS in comparison to human osteoblasts. Anticancer Res..

[51] Park S., Nam J.S., Lee D.R., Kim H., Ahn C.W. (2018). Fetal bovine serum-free cryopreservation methods for clinical banking of human adipose-derived stem cells. Cryobiology.

[52] Liang X., Hu X., Hu Y., Zeng W., Zeng G., Ren Y., Liu Y., Chen K., Peng H., Ding H. (2019). Recovery and functionality of cryopreserved peripheral blood mononuclear cells using five different xeno-free cryoprotective solutions. Cryobiology.

